# Ebbinghaus figures that deceive the eye do not necessarily deceive the hand

**DOI:** 10.1038/s41598-017-02925-4

**Published:** 2017-06-08

**Authors:** Hester Knol, Raoul Huys, Jean-Christophe Sarrazin, Andreas Spiegler, Viktor K. Jirsa

**Affiliations:** 1 Aix Marseille Université, CNRS, Institut des Sciences du Mouvement, UMR 7287 Marseille, France; 2 Centre de Recherche Cerveau & Cognition, Université Paul Sabatier, Université de Toulouse, Toulouse, France; 3 CerCo, CNRS UMR 5549 Toulouse, France; 4 ONERA, Systems Control and Flight Dynamics Departement, Salon de Provence, France; 5 Aix Marseille Univ, INSERM, INS, Inst Neurosci Syst, Marseille, France

## Abstract

In support of the visual stream dissociation hypothesis, which states that distinct visual streams serve vision-for-perception and vision-for-action, visual size illusions were reported over 20 years ago to ‘deceive the eye but not the hand’. Ever since, inconclusive results and contradictory interpretations have accumulated. Therefore, we investigated the effects of the Ebbinghaus figure on repetitive aiming movements with distinct dynamics. Participants performed a Fitts’ task in which Ebbinghaus figures served as targets. We systematically varied the three parameters which have been shown to influence the perceived size of the Ebbinghaus figure’s target circle, namely the size of the target, its distance to the context circles and the size of the context circles. This paper shows that movement is significantly affected by the context size, but, in contrast to perception, not by the other two parameters. This is especially prominent in the approach phase of the movement towards the target, regardless of the dynamics. To reconcile the findings, we argue that different informational variables are used for size perception and the visual control of movements irrespective of whether certain variables induce (perceptual) illusions.

## Introduction

The importance of vision for humans can hardly be overstated: we use vision to guide movements, to identify objects, and to manipulate them. While it is well known that the visual system comprises two anatomically distinct streams, a ventral and a dorsal stream, if and how they function differently has been debated for over three decades. In the early 1980’s, Ungerleider and Mishkin (1982) proposed that the ventral and dorsal stream were associated with processing of ‘what’ and ‘where’ attributes of objects in the visual field^1^. Later, Goodale and Milner (1995) proposed that the ventral and dorsal visual stream are dedicated to vision for perception and vision for action, respectively^2^. Accordingly, allocentric information about an object and its contexts proceeds through the ventral stream and evokes a conscious percept. Egocentric information, in particular information about the location of objects for guiding our movements, passes through the dorsal stream in absolute measures.

The functional dissociation attributed to the two visual streams was originally based on clinical studies. These studies demonstrated that lesions to the ventral stream are uniquely associated with functional deficits, often severe, in reporting physical attributes of various objects while retaining the possibility to manually interact with them. Inversely, lesions in the dorsal stream severely disrupt motion-related information affecting actions. However, ever since Aglioti *et al*.^3^ published their landmark study^3^, visual illusions have become popular to study the proposed dissociation between conscious perception (ventral stream) and (unconscious) perception for the control of movements (dorsal stream) in grasping and pointing tasks. Context-induced visual illusions make targets look smaller or bigger than they are, through for example small context circles around one big ‘target’ circle (i.e., the Ebbinghaus figure). If a strict functional dissociation between the ventral and dorsal stream exists, then conscious perception should be related to the relative size of an object, whereas actions should be affected by the absolute size of an object. In other words, conscious perception is thought to be sensitive to visual illusions. In return, movements guided by egocentric information should be unaffected by visual illusions.

Several studies, both in the context of grasping and pointing, have provided evidence in favour of a functional dissociation between the ventral and dorsal stream^3–7^. In these studies perception was affected by the illusion, but the grip aperture^3–5, 7^ and movement time remained unchanged^8, 9^. Others, though, found that perception and action were equally affected by visual illusions and thus concluded that the same representation of object size guides perception and action^10–15^. These contradictory findings and interpretations are opposed by authors claiming that the illusion effects do not depend on whether the task is perceptual or motor, but rather on the spatial attributes that are used to execute a task^16, 17^. For example, in the same movements the lift and grip force are sensitive to a size illusion, but grip aperture is not^18, 19^. Yet another view was forwarded by Glover^20^, who proposed that visual illusions affect the planning of actions, but not their on-line control^20^. Since experimental support exists for each of the aforementioned approaches, numerous methodological differences suggest that a clear interpretation of the repeatedly contradictory results will be impossible unless systematic and well-parameterised experimental studies disentangle the role of the visual system in perception and action.

One task that has been implemented to test whether the visual system is functionally dissociated, is Fitts’ task^9, 15, 21, 22^. In a Fitts’ task, a participant is asked either to move a stylus on a tablet from a start position to a given target of width (*W*) and distance (*D*) (i.e., single or discrete movement) or to cyclically move between two targets of width (*W*), which are separated by a distance (*D*). By systematically varying *D* and *W*, Fitts^23^ linearly related the movement time (*MT*) to the ratio of movement distance (*D*) to target width (*W*) through the index of difficulty *ID* = log_2_ (2*D*/*W*) by *MT* = *a* + *b ID*. The index (*ID*) expresses the difficulty of the task in bits^23, 24^. The robustness and insensitivity to experimental contexts of this so-called Fitts’ law, as well as it quantitative nature, render Fitts’ paradigm a powerful tool to investigate the dissociation of visual streams.

The question arises whether the linear relationship between the *MT* and the *ID* is affected by a visual context. It is unknown what the effect on the *MT* is when the subjective target size (i.e., perceived *W*) is smaller, or bigger, than it physically is. Therefore, Ebbinghaus-like figures have been implemented in Fitts’ tasks to investigate the effect of perceptual illusion on motor behavior^8, 9, 15, 21, 25, 26^. The results of these studies, however, show many contradictions.

Van Donkelaar^15^ was the first to find an effect of the Ebbinghaus illusion on discrete movements in a Fitts’ task, by showing an increase in *MT* when the targets looked smaller (or at least, were thought to have looked smaller; see below). Later, Fischer, however, failed to reproduce these findings in a similar task and reported an insensitivity of the movements to the Ebbinghaus-like figure^8^. Up to now, to our best knowledge, Fischer’s study remains the only discrete Fitts’ task that failed to show an effect of visual illusions on movement time. Indeed, several studies found that pointing movements were affected by the visual illusion (see Table 1 for a summary of the results of these studies). In one study movements were found to be faster towards bigger looking targets^21^. This contrasts yet another study that showed *MT* to remain unaffected by a combined Ebbinghaus-Müller-Lyer figure. However, precision and amplitude for a certain effective *ID* resembled the perceived *ID* in a discrete Fitts’ task^9^. Thus, the discrete pointing tasks have rendered ambiguous results.

**Table 1:** Effects of Ebbinghaus-like figures on size perception and duration of pointing movements.

	Feedback	Target (mm)	Delay (ms)	Perception (%)	Relative *MT* (%)	Protocol
***Discrete movements***						
Van Donkelaar^15^	OL	30	—	—	S: 94%, B: 100%** (control: 318 ms*)	a
Fischer, exp 1^8^	CL	12	—	**S: 100, M: 96, B: 98** (control: 12.2 mm)	S: 105.6%, M: 104.2% B: 106.4% (control: 359 ms)	b
Fischer, exp 2^8^	LV	12	650	S: 100, B: 95	S: 100%, B: 102.5% (control: 437 ms)	b,f
Handlovsky^21^	OL	50	—	**S: 107**, B: 98 (control: 51.0 mm)	**S: 94–96%**, B:95–99% (control: 464 ms)	c
Alphonsa, exp 1^*^^9^	CL	19	5000	**S: 110**, B: 96 (control: 17.9 mm)	*p* > 0.05, no values reported	d,e
***Reciprocal movements***						
Ellenbürger^25^	CL	14; 40	—	—	**S: 575**/**655 ms, B: 625**/**690 ms**	a
Alphonsa, exp 2*^9^	CL	19	—	**S: 110**, B: 96 (control: 17.9 mm)	not significant; no values reported.	d

All illusion effects are relative to the control condition in percentage if the control condition was present, and marked bold if significant at the *p* < 0.05 level. Else, the values in mm or ms were reported. The illusion conditions consist of Ebbinghaus figures with a small (S), medium (M), or big (B) context size. The visual feedback is classified as open-loop (OL), closed-loop (CL), and limited vision tasks. *The exact *MT*s are not reported. **The *MT*s are not related to a control condition in the study. Experimental conditions: (a) simultaneous presentation of a target with a small context on one side, and a big context on the other side, (b) only the target is surrounded by contexts, (c) the home position as the target could be surrounded symmetrically and asymmetrically by a small, big, or no context size, (d) the symmetric display of the left and right target with, or without context circles, (e) display offset and movement onset after time delay, (f) movement onset after delay.

Next to discrete Fitts’ tasks, reciprocal Fitts’ tasks have also been combined with visual illusions (see Table 1). In a reciprocal aiming task with Ebbinghaus illusions, a longer *MT* and dwell time was reported for big context circles relative to small context circles (note that the authors did not report a control condition)^25^. This result is supported by a different size illusion (i.e., the Müller-Lyer trapezoids) that was implemented in a reciprocal tapping task for which *MT* increased and the endpoints of the movements were more tightly distributed when the target looked smaller^22^. However, in yet another study, *MT* and accuracy measures appeared insensitive to the combined Ebbinghaus-Müller-Lyer figure^9^. Thus, ambiguity in the reported results in the reciprocal Fitts’ task lines up with that in the discrete task version.

The ambiguity in the reported results may well be traced back to methodological differences. Fischer (2001) reported perceptual illusion effects ranging from −0.3 mm to +0.2 mm relative to physical target size. This leaves the discussion open as to whether illusion effects are big enough to identify changes in movement since the perceived *ID* (between 4.09 and 4.15) hardly changes. Van Donkelaar^15^ did not quantify the perceptual illusion effect, so that a lack of illusion and its effects in some of the parameter combinations cannot be excluded, which may explain why he only found significant results for the ‘looking smaller’ condition^27^. Also, different stimulus presentation protocols were applied in the studies in which the Ebbinghaus figure was used to make targets look bigger or smaller. In some experiments, participants were asked to move from the centre between two targets to one of the two simultaneously displayed targets: small context circles surrounded one of the two targets, and big context circles surrounded the other target^15, 25^. In contrast, Alphonsa *et al*.^9^ used the same target as starting and endpoint. Others displayed only one target with or without surrounding circles^8, 21^. Another factor of concern is related to the visual feedback before and during movement execution. The timing and duration of the visibility of the targets, as well as the visibility of the hand during movement execution differs across the protocols (see Table 1). In some cases, the targets had to be memorized due to a delay between stimulus presentation and movement onset^8, 9^. In other cases, targets were permanently visible^8, 15, 25^, or appeared with movement initiation^21^ (see Table 1). Delayed movements and judgments are more likely to be based on conscious perception (i.e., associated with the ventral stream) than on visuomotor information (i.e., reflected in dorsal stream activity)^28, 29^. Memory based, delayed actions lead to stronger illusion effects^30^. The visibility of the hand during the execution of the task was restricted in some studies (commonly referred to as an open-loop task), whereas others did not constrain the visibility of the hand (closed-loop; see Table 1). The availability of visual feedback during aiming tasks allows for online control of movements, which is ascribed to the dorsal stream. The illusion effects for reciprocal, closed-loop aiming movements (see Table 1) speak against a strict functional dissociation of the visual system. Hence, the contradicting results relative to the ventral-dorsal stream dissociation might be explained in terms of differences in methodology, the lack of perceptual quantification of the illusion effect, and relatively small perceptual illusion effects.

Another source of variation that has, to our best knowledge, not been considered so far is in the type of movements elicited in different experiments. In particular, it might be that the presence (or absence) of an illusion and its effect on movements is restricted to a certain control mechanism governing the movements. Dynamical models have sought to disentangle the changes in the movement organization underlying the *MT* in a reciprocal Fitts’ task^31–35^. By analysing movement kinematics, two types of dynamics have been identified, namely of limit cycle and fixed point^31, 36^. A limit cycle is a closed orbit in the state space, which is spanned by the position of the movement *x* and its change in time d*x*/d*t*. A trajectory on the limit cycle thus periodically returns to its starting point. Therefore, limit cycles are typically used to describe rhythmic activity. A fixed point instead is a location in the state space at which there is no movement, that is, no changes in time, d*x*/d*t* = 0. The behaviour around a fixed point is discrete (depending on the nature of the fixed point, attracting, repelling, or both but in different directions, as in Fitts’ task performance; see 31 for details). Thus, the start position and the target can be ascribed as repelling and attracting for movements in discrete Fitts’ tasks. In a reciprocal Fitts’ task, a sudden transition from limit cycle to fixed point behaviour can be evoked by increasing the *ID* ^31^. In the latter regime, the reciprocal movements are effectively concatenated discrete movements. When the perceived target width differs from the physical width, the question is whether it is the perceived or the actual width (*W*) that governs the *ID* and the corresponding movement kinematics. As the kinetics are distinct, it may well be that the answer to this question lies in the type of movement underlying the task performance. Movements of the limit cycle type can be expected to be less susceptible to visual perturbations such as introduced by the Ebbinghaus figure. Two arguments that support this hypothesis are that the evolutionary older rhythmic movements owe their functional integrity to a large part to body-related information (in particular kinaesthesia and proprioception), whereas the evolutionary younger discrete movements rely in particular on the visual system^37^. A second argument is found in the aiming literature in describing larger effects of reducing the availability of visual information on tasks of high level difficulty (typically associated with fixed-point behaviour) compared to low levels of task difficulty (typically associated with limit cycle behaviour)^38^. To date, however, the control mechanisms governing the movements have not been related to the effects of visual illusions on pointing movements.

This leads us to test three hypotheses:

i) A functional visual-stream dissociation exists. In this case, Ebbinghaus figures that evoke perceptual illusions will neither affect the duration nor the precision of movements.
ii) Vision for perception and vision for action cannot be dissociated, that is, a one-to-one mapping of effects on perception and action exists. In this case, all the factors that influence the perceived target size will influence the movement (i.e., its duration and other features). If a target looks smaller, the movement will be slower (similar as to when the *ID* becomes larger). If a target looks bigger, the movement will be performed faster (similar as to when the *ID* becomes smaller).
iii) Whether the movement is influenced by visual illusions depends on the dynamics (i.e., limit cycle or fixed point dynamics), which govern the movement. More specifically, we anticipated that big target sizes that are associated with limit cycle behaviour would remain immune to the influence of visual illusions, whereas small target sizes that are associated with fixed-point dynamics would be susceptible to visual illusions.

Alternative to these hypotheses, however, one could also consider the possibility that the presence or absence of illusion effects on the movements is independent of the presence of perceptual illusion effects per se, but rather, that movements are affected under certain stimulus configurations but not others^16^. Having said this, we are unaware of studies allowing for the formulation of specific predictions in this regard, however. Nevertheless, please note that the first hypothesis *i* does not rule out the existence of interactions between the two streams, as already pointed at by Milner and Goodale (1992). Functionally, however, these interactions are not such that figures causing perceptual illusions lead to ‘motor illusions’. We will investigate these three hypotheses in the present study to clarify whether and how the ventral and dorsal streams are functionally dissociated. By structurally probing a broad range of parameters that have been previously identified in a visual perception study, we will evaluate movements with classical methods (i.e., that have been traditionally used to analyse Fitts’ tasks), and the underlying dynamics. As visual feedback is thought to favour the dorsal stream processing of information, we implemented the Ebbinghaus figures in a reciprocal, closed-loop Fitts’ task.

## Results

### Fitts’ law – the effect of target size on non-normalized durations

**Figure 1:**
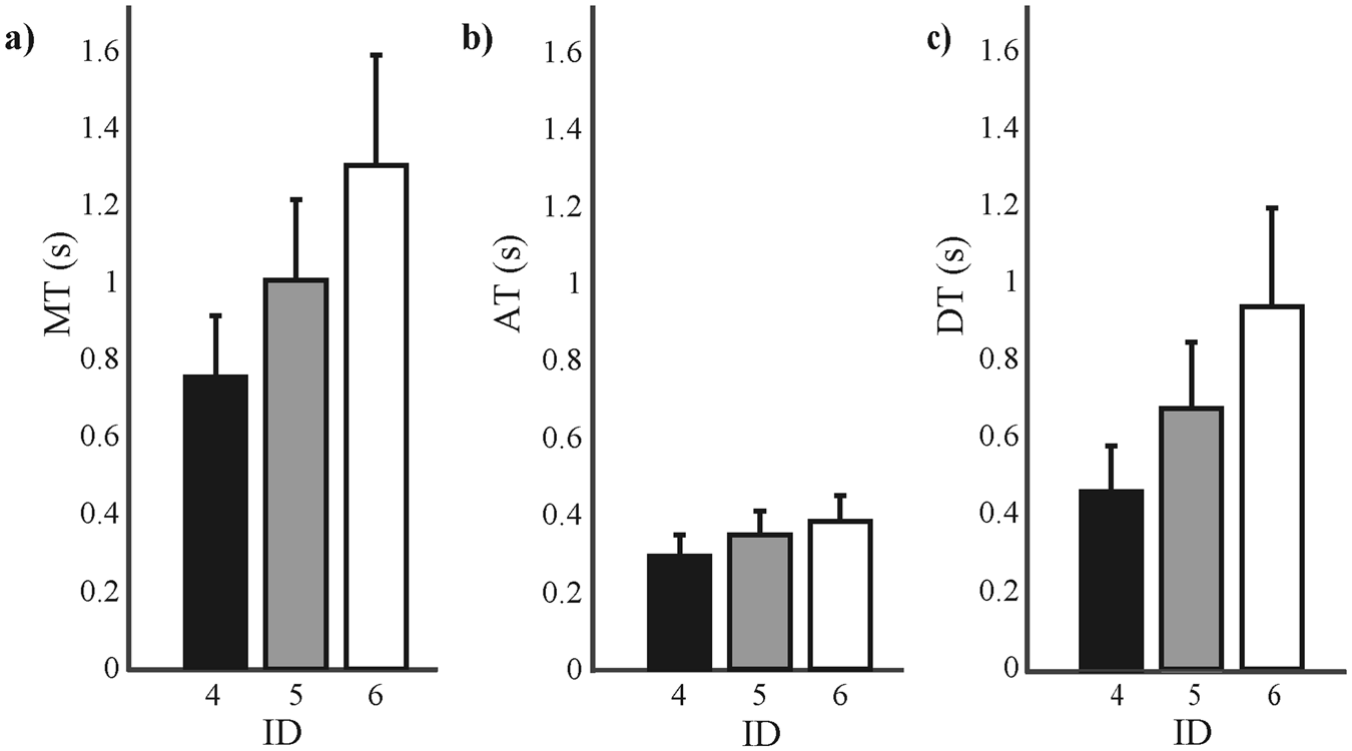
(**a**) Movement time (*MT*), (**b**) acceleration time (*AT*), and (**c**) deceleration time (*DT*) for ID 4 (black), ID 5 (grey), and ID 6 (white). The error bars represent the standard deviation.

We examined how *MT* changed as a result of changing the target size, context size, and the context—target distance of the Ebbinghaus figure (see Methods for more details). The target sizes of 5, 10, and 20 mm corresponded to an *ID* of 4, 5, and 6, respectively. We found that *MT* increased with increasing the *ID* for both the illusion and control trials (for illusion trials: *F*(2,16) = 92.85, *p* < 0.001, *η*_*p*_^2^ = 0.921; Fig. 1a). Thus, Fitts’ law held under both the Ebbinghaus and control conditions. Also, the acceleration time (i.e., the time from movement onset to peak velocity), *AT* (for illusion trials: *F*(1,8) = 50.61, *p* < 0.001, *η*_*p*_^2^ = 0.864; Fig. 1b), deceleration time (i.e., the time from peak velocity to movement offset), *DT* (for illusion trials: *F*(1,8) = 68.36, *p* < 0.001, *η*_*p*_^2^ = 0.895; Fig. 1c) increased with *ID*.

The ratio between the acceleration time and the movement time (*R*_*AT*_/_*MT*_) quantifies the asymmetry of the velocity pattern. The asymmetry has previously been shown to increase (*R*_*AT*/*MT*_ < 0.5) as *ID* increases^33, 34^. In line therewith, the *R*_*AT*/*MT*_ significantly increased as the *ID* decreased (*F*(2,16) = 54.59, *p* < 0.001, *η*_*p*_^2^ = 0.872), which was mainly due to a decrease of the deceleration time as *ID* decreased.

### Illusion effects

**Figure 2:**
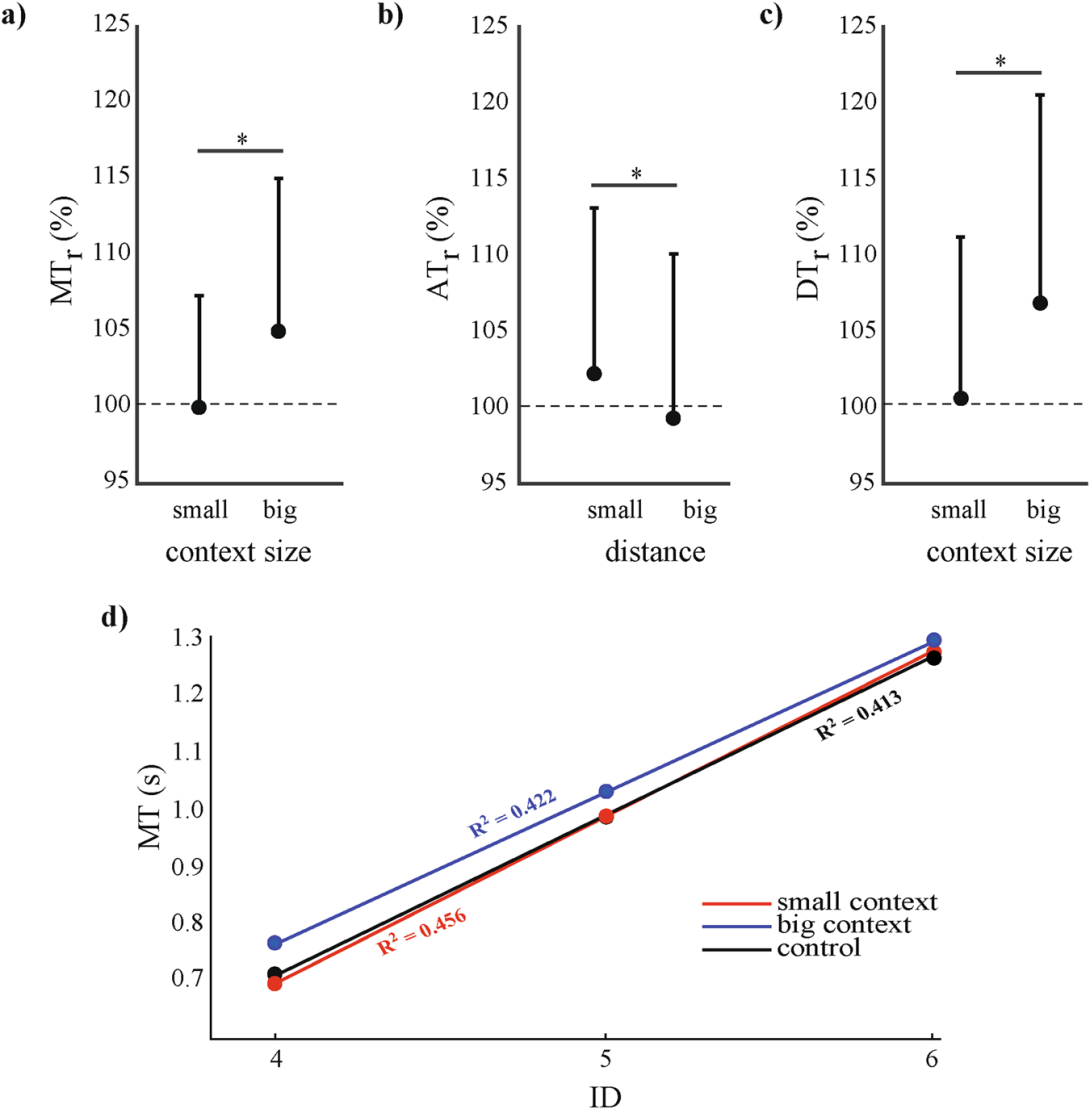
(**a**) The relative movement time (*MT*_*r*_), (**b**) acceleration time (*AT*_*r*_), and (**c**) deceleration time (*DT*_*r*_) as a function of the small and big context circles (**a**,**c**), and target—context distance (**b**). The error bars represent the standard deviation. (**d**) The linear regressions with the corresponding *R* ^*2*^ values of movement time (*MT*) in seconds as a function of the index of difficulty (*ID*) for the small (blue) and big (red) context and the control condition (black).

To answer hypothesis *i* and *ii*, we tested if the Ebbinghaus figure had an effect on various temporal and spatial features of the movements, and which factors determined the observed effects (if any). To control for the effect of target size on various dependent measures we normalized them relative to the control conditions. *MT*_*r*_, *AT*_*r*_, and *DT*_*r*_ signify the *MT*, *AT*, and *DT* relative to the corresponding observed values in the control condition in percentage. Contrasting hypothesis *i*, context size significantly affected the *MT*_*r*_. (*F*(1,8) = 13.97, *p* < 0.01, *η*_*p*_^2^ = 0.640; Fig. 2a); *MT*_*r*_ was significantly bigger than 100% in the big context size condition (*t*(107) = −5.41, *p* < 0.0001). The increase in *MT*_*r*_ could be explained by an increase in the relative deceleration time (*DT*_*r*_) (*F*(1,8) = 17.22, *p* < 0.01, *η*_*p*_^2^ = 0.683; Fig. 2c). The *AT*_*r*_ was affected by the distance between the target and context circle (*F*(1,8) = 5.39, *p* < 0.05, *η*_*p*_^2^ = 0.400; Fig. 2b). On a group level, linear regression on the *MT* over *ID* showed a bigger intercept (*a*) and marginally shallower slope (*b*) for the big context size condition (*a* = −0.28, *b* = 0.26 in Fig. 2d) as compared to the small context (*a* = −0.45 and *b* = 0.29) and the control condition (*a* = −0.39, *b* = 0.28). We additionally performed the regression analyses for each participant individually in order to assure consistency between the here-reported group level and the individual level (see supplementary information).

### Perceptual categories

To further study hypothesis *ii*, according to which a target that looked bigger (or smaller) than it actually was should result in faster (or slower) movements, we identified three perceptual categories based on our recent study on quantifying the Ebbinghaus figure and its effects on perception^27^. The perceptual categories are conditions in which the target circle was perceived as *looking smaller*, *looking bigger*, and *no illusion effect* as compared to the control condition. The *MT* relative to the control condition for the 12 Ebbinghaus figure conditions was divided into these three perpetual categories. These categories identify which illusion figures evoked a significant perceptual illusion effect and identify the direction of the effect (i.e., bigger or smaller). To compare the perceptual effect with the effects of the Ebbinghaus figure on *MT*, we calculated the illusion effects relative to the control condition (in percentage) for both the perception and *MT* data, and then correlated them. The relative MT was significantly bigger than 100% (one tailed *t*(62) = 5.2, *p* < 0.0001) only when the targets were perceived as smaller than they really were (*looking smaller* category, mean = 104.6 ± 7.0). Thus, this analysis did not corroborate hypothesis *ii*.

### Correlation perception and movement time

**Figure 3:**
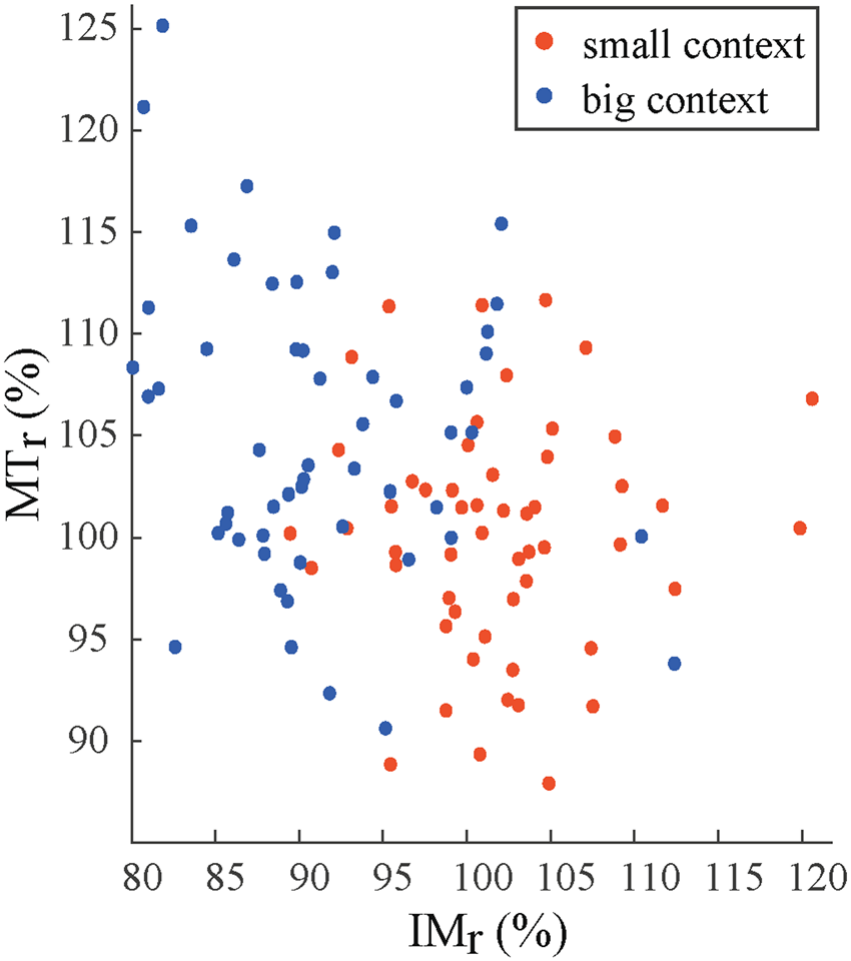
The relative movement time (*MTr*) as a function of relative illusion magnitude (*IMr*; from ref. 27) and context size (for the same participants). Each dot represents a condition for a participant. Red and black dots represent the small and big context condition, respectively.

Since the complete dataset of the illusion magnitude (perception) of the same participants was at hand, we could test whether the perceptual effects correlated with the movement effects. As can be seen in Fig. 3, relative perceived target size correlated negatively with the relative movement time (*r* = −0.32, *p* < 0.001) with a negative slope as indicated by a linear regression analysis (*a* = 127.87, *b* = −0.26); perceived as smaller (larger) targets were accompanied by longer (shorter) relative movement times. Since context size significantly affected *MT*_*r*_, we computed the correlations for both context size conditions separately. Only the big context condition materialized in a significant (negative) correlation between the relative illusion magnitude (*IM*_*r*_) and *MT*_*r*_ (*r* = −0.29, *p* < 0.05, *a* = 126.27, *b* = −0.23; blue dots in Fig. 3), though the correlation for the small context condition was negative, albeit it marginally, as well (*r* = −0.012, *p* > 0.05, *a* = 96.86, *b* = 0.03; red dots in Fig. 3).

### State space analysis

To explore whether the influence of the Ebbinghaus figure on movement is dependent on the movement dynamics that underlie the movements (hypothesis *iii*), we analyzed the state spaces associated with the movements. These spaces are spanned by the horizontal position and its first derivative with respect to time, *x*(*t*), d*x*(*t*)/d*t*. Since the contribution of the movements in the sagittal plane (i.e., away from the body of the participant) on the trajectory length was negligible (see Supplementary Information for further details), we only used the horizontal position data for further analyses (as is typically done^33, 34, 39^). The state space analysis consisted of constructing the vector fields in the state spaces, which describe the change in magnitude and direction at given points in the state space of a system. With respect to the present work, the arrows represent the vectors that indicate the magnitude and direction of the movement’s rate of change at the corresponding points in the state space. Vector fields are the graphical representations of the system’s dynamics^40, 41^ and were here reconstructed from the concatenated horizontal position data to verify whether fixed points were present at the highest *ID*s. Fixed points are points in the phase space at which the system remains unchanged in time, that is, d*x*/d*t* = 0. At a fixed point, the vector has zero magnitude. Around it, however, the vectors are pointing in opposing directions, so that a fixed point is more easily recognisable by a location in the phase space where short arrows are pointing in opposing directions. If indeed a fixed point behaviour is present, the maximum angle (*θ*_max_) between the vectors close to the fixed point can be expected to be bigger than 90°^31, 33^. For limit cycle behaviour, the vectors should point in a similar direction, and therefore the angle should not exceed 90°. The maximum angle (*θ*_max_) was calculated around the end point of the movement^31^ (see Methods). There was no significant difference between the left and right target (*t*(107) = −1.7743, *p* = 0.08). We therefore averaged across both targets for the remaining analyses.

**Figure 4:**
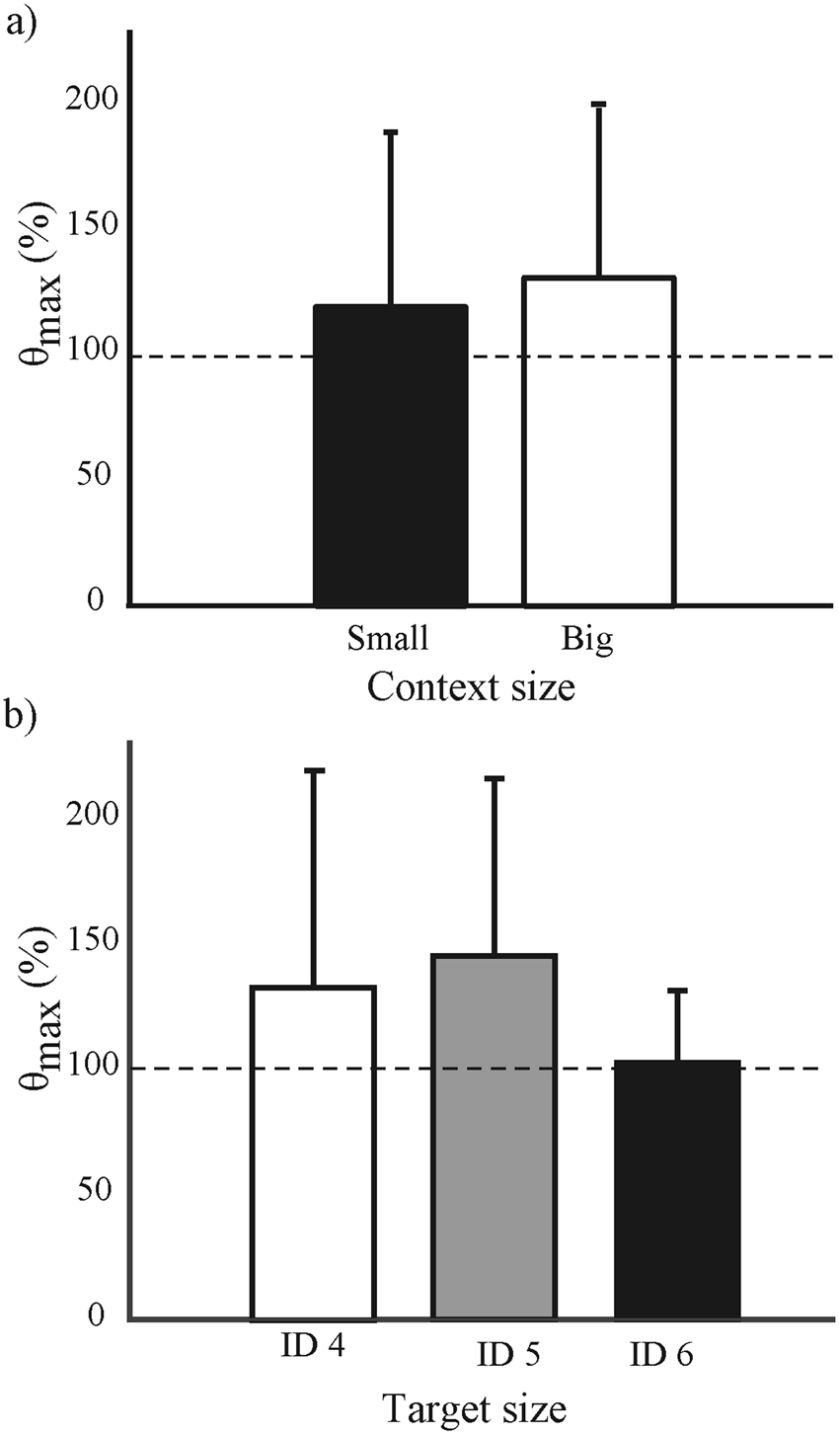
The relative maximum angle between vectors for (a) context size and (b) target size. In panel (**a**) the two bars represent the maximum angle in percentage (*θ*_max_) between vectors in a vector field for the small (black) and big (white) context, relative to the control condition. In panel (**b**) the bars represent *θ*_max_ in percentage for index of difficulty 4 (white), 5 (grey), and 6 (black). The error bars represent the standard deviation.

The absolute mean values for the lowest *ID*, that is, *ID* 4, showed a *θ*_max_ smaller than 90° (77°), whereas those of *ID* 5 (116°) and *ID* 6 (133°) were larger than 90°. These results indicate that the movements at *ID* 4 were associated with limit cycle dynamics while those at *ID* 5 and 6 were associated with fixed-point dynamics. Relative to the control condition, both *ID* and context size affected *θ*_*max*_ (context size: (*F*(2,34) = 4.85, *p* < 0.05, *η*_*p*_^2^ = 0.222; Fig. 4a; *ID*: (*F*(2,34) = 3.35, *p* < 0.05, *η*_*p*_^2^ = 0.165); Fig. 4b). Post-hoc tests indicated that *ID* 6 was significantly different from *ID* 4 and 5 (*p* < 0.01). Subsequently, the *MT* relative to the control condition was divided into a limit cycle (*θ*_max_ < 90°) and fixed-point class (*θ*_max_ > 90°). The *MT* relative to the control condition for the limit cycle class (mean = 101.3 ± 8.8) was not significantly different from that of the fixed-point class (mean = 101.9 ± 8.6; *p* = 0.74).

**Figure 5:**
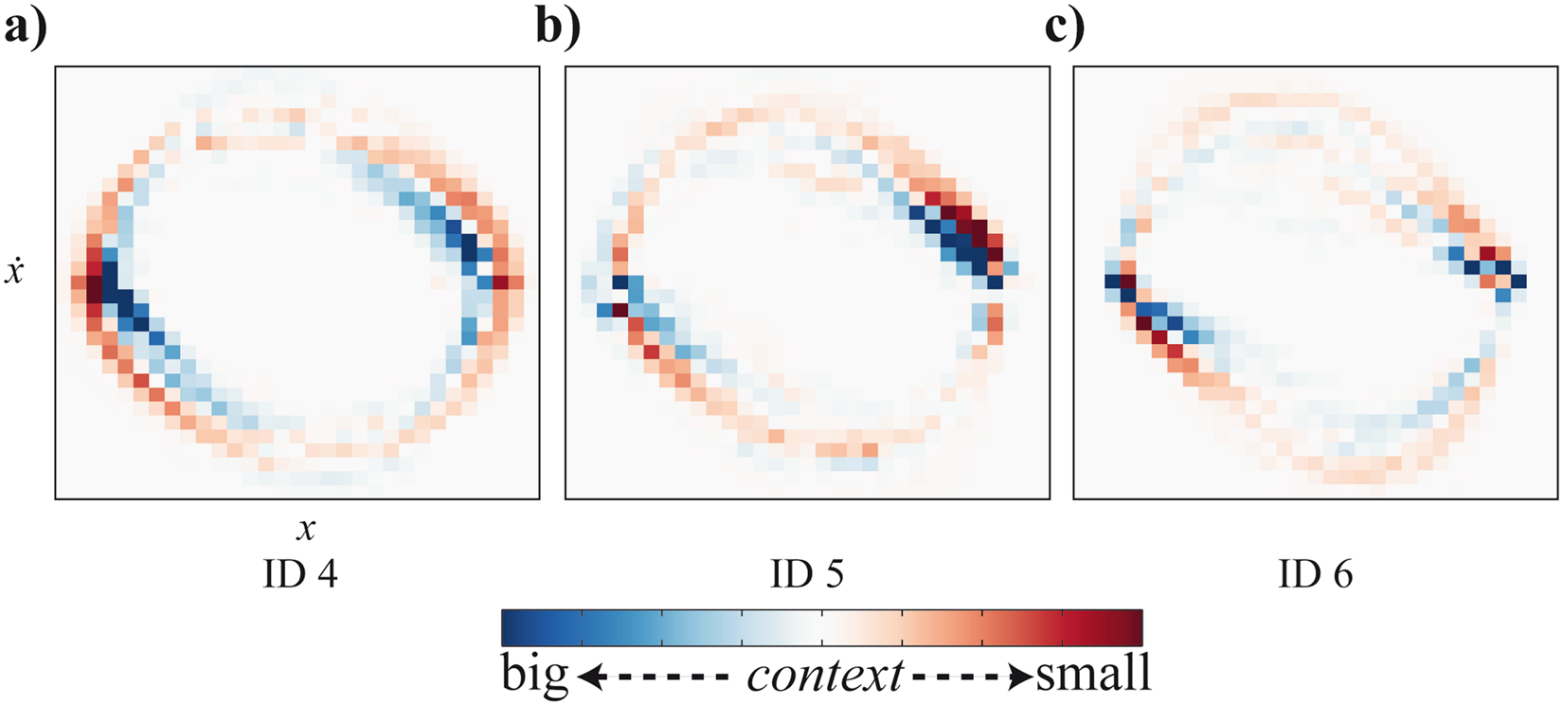
Difference probability distribution for (**a**) ID 4, (**b**) ID 5, and (**c**) ID 6. The red colouring marks a higher probability for the small context condition; the blue colouring signifies higher probabilities for the big context condition.

The movements’ probability distributions in the state space show how long (i.e., how many samples) the participants spent in a given bin, that is, a small region in the state space. The probability to find a participant’s movement in a certain state is specified by counting the samples in the corresponding bin in the state space. The difference probabilities in Fig. 5 show the difference between the probability distributions in the small context condition and the big context condition (from the concatenated movements of all participants).

These difference probability distributions provide information about the movement kinematics and to some extent the underlying dynamical classes that have previously been identified for reciprocal aiming movements^31^. In probability distributions, limit cycle behaviour shows up as circular, more or less symmetrical orbits with probabilities that are fairly uniform^42^. For single aiming movements the symmetry is obtained by an acceleration and deceleration time of similar duration^34^. Fixed-point behaviour typically involves asymmetric velocity patterns with a deceleration (and dwell time) phase longer than the acceleration phase. The probability distributions typically show a peak close to the fixed point^42^. Thus, hints as to which dynamics are adhered to may become apparent (among others) in a difference probability distribution. In Fig. 5, a stronger deviation of a perfect circle, that is, asymmetry for the big context condition (in blue) becomes apparent for *ID* 4 and 5 (Fig. 5a,b, respectively) compared to the symmetric, circular shaped probabilities for the small context condition (in red). It can further be seen that the amplitude for *ID* 4 seems smaller for the big context condition than for the small context condition. For *ID* 6, an asymmetric pattern can be found for both the small and big context (red and blue, respectively). Thus, the big context condition prolonged the phase of movement deceleration as compared to the small context condition, and pushed the participant to perform a sequence of discrete movements rather than a smooth cyclic movement between the two targets. Thus, although we found some changes in the phase space when varying context size, we did not find evidence that the illusion effects were dependent on the movement type, so that hypothesis *iii* was not confirmed.

### Classified movement endpoints

The analysis of the classified movement endpoints (into misses, one-sided or two sided overshoots where the target is traversed once or twice, and the valid movements) showed that target size affected almost all the classes (except the misses). Bigger targets were associated with a larger amount of valid movements and less overshoots (i.e., one-sided and two-sided). No significant effects of context size, or context—target distance were found (see Supplementary Information for further details).

### Endpoint distribution

**Figure 6:**
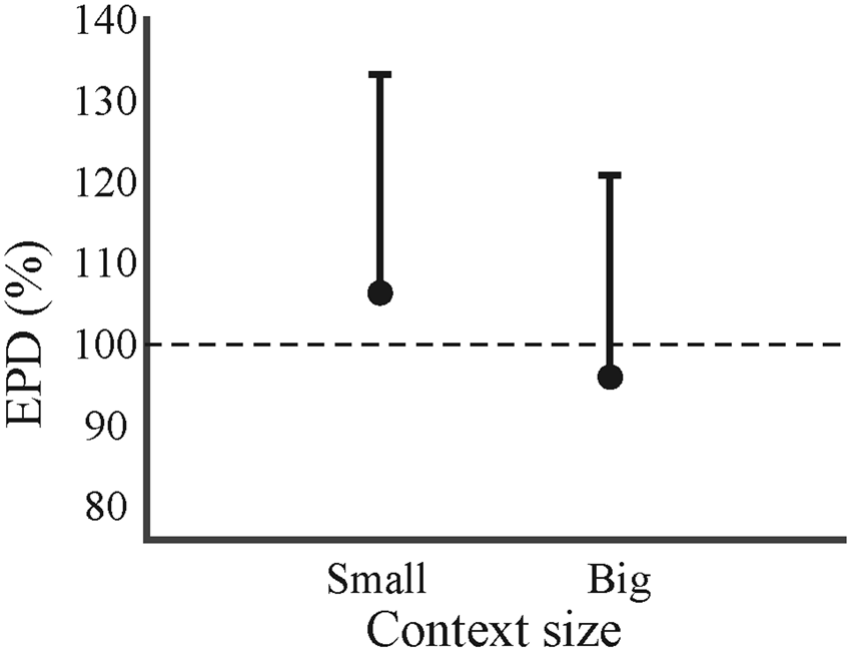
The endpoint distribution (*EPD*) in percentage for the small and big context, relative to the control condition. The error bars represent the standard deviation. The dotted line signifies the EPD of the control condition.

The endpoint distribution (*EPD*; see Methods, equation 1) captures the width of the movements’ endpoints. The first eigenvector was significantly affected by context size (*F*(1,17) = 27.839, *p* < 0.001, *η*_*p*_^2^ = 0.621): a small context increased the *EPD* on average with 7%, whereas a big context decreased the *EPD* on average with 2.7% relative to the control condition (i.e., an *EPD* of 5.89, 9.19, 15.72 mm for the small, medium, and big target respectively; Fig. 6). With respect to the actual target size, a small context reduced the endpoint distribution with 1.06 mm and a small context circle with 1.96 mm. Thus, a small context size makes participants use more space in the target, while a big context size makes the endpoints more focal. However, the correlation between the illusion magnitude (perception) and the *EPD* failed to reach significance (*p* = 0.22, *r* = 0.12).

## Discussion

The literature on (pointing) movements to size-contrast illusions (using, for example, the Ebbinghaus figure or the Müller-Lyer trapezoids) is filled with contradictory results. The experiment reported in this manuscript was especially designed to systematically examine the effect of various Ebbinghaus figure configurations on reciprocal, closed-loop aiming movements. We identified three hypotheses, each of which we discuss in the following with regard to the literature and reported experimental findings.

*Hypothesis i*. A functional visual-stream dissociation exists. In this case, Ebbinghaus figures that evoke perceptual illusions will neither affect the duration nor the precision of movements.

If the anatomical dissociation between the ventral stream and the dorsal stream would indeed be associated with a dissociation of function as Milner and Goodale proposed^2, 43^ and provided evidence for^3, 6^, movements towards visual illusion figures would be insensitive to their illusionary (perceptual) effects. Our findings demonstrate that mainly one of the Ebbinghaus figure parameters (but not the other two) influenced the movement’s temporal and spatial (precision) features. They consequently speak against a strong and consistent functional dissociation of the visual system. These findings are the more surprising because dorsal stream activity is commonly related to the online control of movements^44, 45^, and especially to reciprocal aiming movements, which require continuous and direct visuomotor transformations^22^ as compared to discrete tapping/pointing. Thus, if in any case, the proposed insensitivity of movement to visual illusions should especially surface for reciprocal movements.

Little is known about the influence of size-contrast illusions on reciprocal, closed-loop aiming movements as in a Fitts’ task, however. The few reported results on this subject are contradictory. For example, in the study of Skewes *et al*.^22^, the Müller-Lyer trapezoids affected the perception, the *MT*, and the movements’ precision^22^. However, in another study, the combined Ebbinghaus-Müller Lyer figures did affect the perception but neither the *MT*, the amplitude nor the precision in reciprocal tapping^9^. In the latter study, however, the movement amplitude and the radial error of discrete tapping were susceptible to the perceived target size and perceived distance. Regardless the different protocols that were applied (i.e., the way authors implemented the visual illusions, quantified perception, and tested parameters), the common denominator for these reciprocal, closed-loop aiming studies is that they speak against a strict functional dissociation of the visual system.

In return, Skewes *et al*.^22^ pointed out that the illusion effects on movements that were reported by Franz (2001) and Van Donkelaar^15^ might have been due to the absence of visual feedback of the moving arm^2, 22^. That is, in the absence of this feedback, visual information is most likely processed in the ventral stream since the memory trace in the dorsal stream is short-lasting as compared to the trace in the ventral stream (i.e., visual memory)^46^. This was supported by empirical evidence showing that when visual feedback was introduced during the task, an effect of the illusion on pointing^8^ and grasping^7^ was not found. Our results, however, showed that the execution of the perceptual-motor task with visual feedback was consistently influenced by the visual illusion for one Ebbinghaus figure parameter (out of three). This finding is in accordance with Skewes *et al*. who conducted a closed-loop reciprocal tapping experiment with Müller-Lyer trapezoids, and found that both the *MT* and the precision were affected. Hence, it seems unlikely that differences in the ventral stream or the dorsal stream memory traces can account for the presence (or absence) of a ‘motor illusion’.

Besides the studies that incorporated visual illusions, several studies have investigated the effect of visual feedback in Fitts’ tasks by changing the mapping between the hand movement (effector space) and the cursor movement on the screen (task space)^47–49^. These studies showed that movements under visual feedback of the cursor movement in a particular nonlinear mapping with the hand movement were faster and more accurate than with a proportional mapping of cursor and hand movement. Although Fernandez and Bootsma (2004) argued that the visual feedback would be more effectively used because the cursor is moving slower when it is close to the target, Brenner and Smeets (2011) suggested that participants moved faster than without a nonlinear mapping of the cursor because they missed fewer targets (i.e., they traded off accuracy for speed). Following the latter line of reasoning, the participants might have moved slower when the target appeared smaller because they had more difficulties hitting the targets. However, the number of invalid movements (the total of misses, undershoots and overshoots; see supplementary information) remained unaffected by the illusion in our experiment. Thus, it seems more likely that the velocity close to the target assured a successful hit of the target.

*Hypothesis ii*. Vision for perception and vision for action cannot be dissociated, that is, a one-to-one mapping of effects on perception and action exists. In this case, all the factors that influence the perceived target size will influence the movement (i.e., its duration and other features).

Franz and colleagues^10, 14^ proposed that the same visual representation underlies the illusion effect in perception and in action. That hypothesis finds support only if the factors that influence the perception are the same as the ones that influence the movement. The results presented in this work do not support this hypothesis: We showed that both *MT* and *DT* increased for ‘looking smaller’ targets surrounded by big context circles. This was not true for ‘looking bigger’ targets surrounded by small context circle. In accordance with this finding, context size has been found to affect the *MT* towards Ebbinghaus targets in pointing tasks^15, 21^. In a previous study, we showed that the target size, the context size, as well as the distance between the context circles and the centre of the target might all contribute to the illusion magnitude (see ref. 27). Here, we show that only one parameter (context size) out of three that affected visual perception, consistently affected the movement (i.e., mainly context size affected *MT*, *DT*, *EPD*, *θ*_max_). It should be noted, however, that the selected subset of conditions as compared to ref. 27 might have masked an effect of distance. Furthermore, a comparison between performances on tasks that are quantified via different measures, with in all likelihood unequal precision to pick up performance differences should be treated with care. At the same time, in ref. 27 all three parameters were found to affect perception whereas in the present study the same factors (although a smaller range for the tested factors was selected) did not affect movement. Given that it is unlikely that measurement precision is affected by how the Ebbinghaus figure is parameterized, we are quite confident in stating that our findings cannot be explained by the theory that perception and movement are governed by the same visual information^10, 14, 50^.

Regardless the factors that determine the effect of the illusion on movement and perception, the question remains whether the movement is scaled according to the perceived target size (as predicted by hypothesis *ii* here above). Van Donkelaar^15^ suggested that the relative size of the targets determined the *MT* rather than the absolute size, by showing that movements towards perceptually smaller targets were significantly slower than to perceptually bigger targets. However, as said, in that study the perceptual illusion effect was unfortunately not quantified, that is, the *MT* was scaled with the (or: a supposedly) perceived target size^15^, see also ref. 25. Handlovsky *et al*.^21^ also showed a decrease of *MT* when the target looked bigger, however, this effect was asymmetric; participants did not move slower when the target was surrounded with big context circles. This asymmetry might be explained by the lack of illusion effects for the big context size condition, because the experimental conditions failed to make a target appear smaller than it was. In the previous study in which we quantified the illusion magnitude of the Ebbinghaus figure on visual perception, we demonstrated that different experimental conditions could make a target look bigger and smaller. The results of the present reciprocal pointing task, however, demonstrated only an increase in *MT* when the target looked smaller, and therefore a unidirectional effect in the opposite direction as in the discrete aiming movements in Handlovsky *et al*.^21^.

The unidirectional effect might seem to contradict the correlation that we found between the perceptual illusion magnitude and the *MT* (for the given data range). This can be interpreted as if movements would be affected by the perceived target size, as Van Donkelaar^15^ suggested, and therefore support hypothesis *ii*. However, the small correlation reported in our study was mainly explained by the big context condition. Although we found a small correlation with a relatively small sample size, the correlation that we found between perception and action is in accordance with similar (low) correlations in grasping^51^. Taken together, our results partially confirm that the *MT* is scaled according to the perceived target size; the scaling was present (only) when the target looked smaller. Because the perceptual illusion effect was not quantified in Van Donkelaar^15^, and Handlovsky *et al*.^21^ failed to evoke a ‘looking smaller’ illusion, it cannot be excluded that these studies would confirm our findings under systematic parameter variations of a wider range of illusion evoking parameters. Whether or not movement adaptations scale in accordance with the perceived target size (when they do) remains an open question, however.

*Hypothesis iii*. Whether the movement is influenced by visual illusions depends on the dynamics (i.e., limit cycle or fixed point dynamics), which govern the movement.

Continuous reciprocal aiming has been extensively analysed with regard to the underlying dynamics^31, 34–36^. Under low accuracy constraints, movements have been shown to be continuous and governed by limit-cycle behaviour. Under high(er) accuracy constraints the movements are governed by fixed point behaviour (i.e., moving between two fixed points, which are (located at) the targets)^31, 52^. Following previous studies^31, 52^, we expected the transition to happen around *ID* 5, and hence we selected *ID* 4, 5, and 6 in our present experiment. If the perceived target size is driving the reciprocal movement, the underlying dynamics should correspond to those associated with the perceived target size instead of the real target size. The increase in both *DT* and *MT* together with a bigger maximum angle between the vectors around the endpoint of the movement for the lower *IDs* due to the influence of the big context size hinted at changes in the underlying movement dynamics. These changes, however, were not indicative for a transition from limit cycle to fixed-point behaviour. Moreover, stronger asymmetries in the state space were seen for each *ID* due to the introduction of a big context size. Furthermore, illusion effects on *MT* did not differ for classified limit cycle and fixed-point behaviour. That is, movement adjustments were not dependent on the motor class utilized. This leads us to conclude that if movements are affected by visual illusions, they are so irrespective of the dynamics that govern these movements. This conclusion stands in contrast to our expectation, namely that the Ebbinghaus figure would have less influence on fairly uniform cyclic movements as compared to a sequence of discrete movements. The expectation was based on a phylogenetic argument and the experimental finding showing that vision is of less importance in conditions demanding little accuracy than in stringent accuracy-constrained Fitts’ conditions^38^. We tentatively propose that our expectation was wrong because in the present task context, while the precision constraints were scaled by the task, in all conditions the movements had to be made relative to a precisely defined part of the workspace. That is, (spatial) drift is not compatible with successful task performance, the prevention of which requires visual monitoring (be it continual or intermittent).

These findings allow for a discussion of the changes in behavioural dynamics due to the introduction of context circles around a target. To give a mechanistic explanation, we will reflect on the merit of fixed points and of limit cycles. As we described in the Results section, limit cycles emerge in nonlinear systems and describe a cyclic movement, whose flow is in particular not governed by fixed points. Inside any closed trajectory (e.g., limit cycle) there is at least one fixed point see, e.g. ref. 53. Fixed points are locally well defined in state space. With respect to aiming movements, Mottet and Bootsma (1999) proposed a Rayleigh-Duffing model to reproduce the kinematic patterns. In that model, the fixed-point is attracting when the participant is aiming at it, but repelling when the participant is departing from the target. This fixed-point is called a saddle. The movement between two targets is due to the link between the repelling force from one target with the attracting force from the other target, and vice versa (which is a so-called heteroclinic orbit). Within this orbit a limit cycle emerges around the fixed point that is an unstable spiral. As a consequence, the changes in behavioural dynamics can result from three changes: first, the target width and its context (i.e., Ebbinghaus figure) directly affect the location and the stability of the (saddle) fixed point. Second, the Ebbinghaus figure changes the stability and its location of the unstable spiral that is located between the two saddles. And third, the context circles of the Ebbinghaus figure enrich and hence alter the landscape of attractors (e.g., fixed points, limit cycles). All the three mentioned considerations could cause quantitative changes (e.g., *MT*) but also qualitative changes (called bifurcations such as the transition from discrete fixed-point behaviour to cyclic limit cycle behaviour).

For the latter option, one explanation could be the addition or change of stability of anchor points. Anchoring is the deformation of the phase flow associated with a reduced spatio-temporal variability, which is often present at particular regions of the phase space (generally around the movement endpoints)^54–58^. Around anchor points, critical task-specific information is available for the organization of behaviour^54^. In the study of visual information on cyclic arm movements it was found that fixating the gaze at movement endpoints actively created anchor points^58^. Anchor points may also be imposed through constraining gaze to particular regions^57^. The theoretical work of Jirsa *et al*.^31^ showed anchoring and the influence on coordination patterns with external information. The effect of external driving directly affected the system’s state variables, that is, multiplicative coupling. Thus, potentially the introduction of Ebbinghaus figures can create anchor points, or change its strength. More specifically, the context circles can serve as anchor points for the visual gaze for which visual perception and action are linked through multiplicative coupling terms. This hypothesis, however, remains to be tested.

Next to the three hypotheses discussed above, our findings bear on alternative views found in the literature, one of which we formulated following the hypotheses, which we will discuss here below. For instance, in the planning-control model, proposed by Glover^20^, the planning of a movement is thought to be susceptible to the visual illusion, but its control is not. The planning, but not the control component is small in reciprocal tapping movements, which therefore supposedly resist the visual illusion. However, this explanation cannot account for our findings of an affected *MT* and *DT*, maximal vector field angle, and endpoint distribution. Moreover, the effects of the illusion-based pointing movements were mainly present in the deceleration part of the movement (i.e., *DT*). *DT* is typically associated with online control^59^, and has been shown to be susceptible to visual illusions^21^. Therefore, the evidence presented here is at odds with the planning-control model.

In different studies, authors have considered the specificity of dependent variables to assess the effect of visual illusions on conscious perception and action. For example, it has been shown that grip aperture, that is, a measure widely used to highlight the effects of visual size illusions on action^3, 4, 14^, is adjusted at the very beginning of movement onset based on the position of the grasp points on the object rather than the distance between them^18, 19, 60, 61^. Thus, the finding that some aspects of action are somewhat resistant to size illusions may reflect a dichotomy between the processing of visual information for different spatial attributes (e.g., size and position), rather than between perception and action^18, 19, 61^. Our results show similarities with Smeets *et al*. (2002) who concluded that: “The illusions affect some aspects of spatial perception. Whether this affects execution of a task does not depend on whether the task is perceptual or motor, but on which spatial attributes are used in the task.” Along similar lines we suggest that whether a task (perceptual or motoric) is sensitive to Ebbinghaus figures may well depend on the (type of) information that is being used for that given task. That is, whether and which task parameters result in an illusion (perceptual, motoric) depends on the information used for task accomplishment rather than whether the task is perceptual or motoric.

Several potential methodological pitfalls have been identified in previous studies on the effects of size illusions on perception and/or action. As for example, the comparison between the perceptual illusion magnitude and the perceptual-motor illusion magnitude was previously not made due to the differences in measurement units (i.e., distance versus speed)^22^. Therefore, the data presented here are reported relative to within-participants’ control conditions (in percentage), and the data for perception (retrieved from ref. 27) and the Fitts’ task were matched per participant. The comparison between studies needs to be handled with care, however. Clearly, the perception task, and its quantification are not identical, and cannot be identical, to (that of) the perceptual-motor task. The perceptual staircase procedure that was used to quantify the perceptual illusion magnitude in ref. 27 consisted of a probe that was scaled according to the (binary) responses of the participant on one side, and an Ebbinghaus figure on the other side that was kept constant within one staircase^27^. The Fitts’ task was designed to be symmetric by presenting two identical Ebbinghaus figures (and not a participant-adjusted probe on one side and an Ebbinghaus figure on the other side) on the left and right side of the screen to avoid task-induced asymmetries between left-right and right-left movements, and to allow for inter-subject comparisons.

Next to this procedural issue, and probably more important, it is clear that the quantification of the perception and the movements are different (with that of the movements arguably being far more precise than that of perception). One may therefore indeed question whether inferences based on the comparison between the data of a perceptual study^27^ and the present study are valid. We believe they are for the following reason. The results of the perceptual study^27^ indicated that the measurement precision sufficed to find effects for all three Ebbinghaus figure parameters. In the present movement study, we consistently found significant effects on movement for one parameter (context size) but not for the other two parameters (target size and target – context distance). As measurement precision cannot be assumed to depend on the parameter tested for, we may safely conclude that the movement measurement precision was sufficient to detect effects if present. Our failure to detect effects of the other two parameters must thus imply that these parameters did not influence the movements. Given that insufficient measurement precision in both studies can be ruled out to have impacted our pattern of results, we are confident that our inferences based on the comparison between the two studies hold.

Another issue that potentially may have impacted our results is that of obstruction avoidance. Some authors have discussed whether the context circles of the Ebbinghaus figures might be identified as obstructions that should be avoided by the system^62^. By fixing the coverage of the circumference by the context circles to 75%, and thus the open space to 25%, we tried to keep the obstruction of the path towards a target equal over illusion conditions. If obstruction avoidance had been triggered by the size of the obstructers, in our case the size of the context circles, then the bigger context size would have obstructed more than the smaller context circles, and small context circles more than the control condition (i.e., no context circles). Furthermore, the distance between the target and context circles should have influenced the illusion effect on movement as was shown for grasping movements^4^. We did not find evidence in that regard, and therefore assume that a different mechanism is responsible for the illusion effects on the perceptual-motor system. Note that our believe that obstruction avoidance has played a negligible role, if any, in our present study is supported by a large, multi-lab study of Kopiske *et al*.^51^ that examined the obstruction avoidance hypothesis in 144 participants. They found no evidence that the effects of visual illusions on grasping could be explained by obstruction avoidance. We therefore are confident that our reported effects cannot be traced back to obstacle avoidance.

In this work we developed a method to assess the movement on the transverse plane (i.e., spanned by the lateral *x* and anterior-posterior position *y*). This resulted in a detailed classification of targeting and movement. The reasoning for this assessment of movements on the plane is twofold. Firstly, the targets are of circular shape, which gives a restriction in all directions on the plane (e.g., compared to elongated target shapes such as bars). The target shape (e.g., squared, circular, diamond, and triangular) has shown to affect movement time in Fitts’ tasks^63^. This experimental result may indicate not only a change in movement in the horizontal direction (i.e., shortest path between the targets) but also an involvement of the anterior-posterior direction. In other words, the length of the trajectory is longer than the shortest path depending on the target shape. This means that analysing the movement in the horizontal direction (projection of the movement on the *xy*-plane onto the lateral direction *x*) may not always be justified. Secondly, the effect of the Ebbinghaus figure on movements was expected to be quantitatively small. In a previous study we have shown that perception of the same Ebbinghaus figures resulted in small illusion magnitudes (rarely up to 10% of the target size)^27^. It was therefore unclear whether the effect of the Ebbinghaus figures could be measured from the displacement in horizontal direction, or that both directions were required. Because of these two points, the target shape effect on movement and the expected small effects that we aimed to quantify in the movement, we considered the actual trajectory on the plane. Thus, the here presented method allows for a detailed analysis of aiming movements, which especially suits experiments working with (visual or motor) perturbations.

We found unambiguous evidence that variations in one Ebbinghaus figure parameter (context size), similar to scaling the perceptual illusion effect, consistently affected the pointing movements. Therefore the hypothesis that the visual streams are functionally dissociated is not supported. Variations in the other figure’s parameters that elicited perceptual illusions in the same group of participants did not (or hardly) affect the movement (target size affected the maximum angle in the vector fields). That is, we neither found evidence for the hypothesis that perception and action are guided by the same internal representation (nor for the hypothesis that the occurrence of ‘motor illusions’ depends on the motor class underlying the behaviour). Can these findings that lead to opposing interpretations as to the validity of the hypothesis that the visual steam is functionally dissociated be reconciled by acknowledging, as Milner and Goodale^43^ did, the existence of cross talk between the ventral and dorsal stream? One would expect that interactions between the streams could attenuate the effect of a functional segregation. To reconcile the present results via stream interaction requires that the (degree of) interaction depends on the parameters via which the (perceptual) illusion is brought about. This would imply that the visual system would be sensitive to these at an early stage of the visual processing, which we deem unlikely. At least, we are not aware of research pointing into that direction. This issue, however, cannot be answered with purely behavioural studies but requires the utilization of high-resolution brain imaging techniques. Regardless, whether the geometry of a visual figure elicits a motor illusion appears to be independent of whether it elicits a perceptual illusion. These findings, which cast doubt on the assumption that visual illusions are an appropriate means to study the supposed functional dissociation of the visual system, can be explained by assuming that which informational variables are extracted from a geometrical outlook depends on the task, including whether it is perceptual or motoric. It remains to be discovered which anatomical regions are organized functionally in the execution of (visually perturbed) perception and action tasks using high resolution imaging techniques.

## Methods

### Participants

Nine (self-declared) right-handed participants (5 females; age 29.5 ± 3.7 years) who reported having normal or corrected to normal vision volunteered in the experiment. The participants were naïve to the purpose of the experiment. This study was approved by the local ethics committee (CPP Sud-Méditerranée I) and was in accordance with the Helsinki Declaration. All participants gave a written informed consent prior to their participation.

### Apparatus

The participants performed aiming movements between two targets with a hand-held stylus (18 g, 156.5 mm long, ø 14.9 mm, ~1 mm tip) across a digitizer tablet (Wacom Intuos XL; with a resolution of 200 lines per mm (5080 lpi)). The tip of the hand-held stylus was represented by a red dot on the monitor (Dell P2714H with a size of 597.9 by 336.3 mm (1920 × 1080 pixels) that displayed 60 frames per second. Position time series were acquired from the tablet via custom-made software (250 samples per second). The targets were displayed and designed using the Psychophysics Toolbox^64, 65^ in Matlab R2014b (The MathWorks Inc., Natick, MA). Filled black circles were presented against a white background and multisampled (open GL) to control for aliasing effects. The participants sat at a 60 cm distance from the monitor (establishing a viewing angle of 52.96° × 29.27°) and their head was supported with a chin-rest so as to ensure that the distance between the head and the monitor remained fixed.

### Procedure

**Figure 7:**
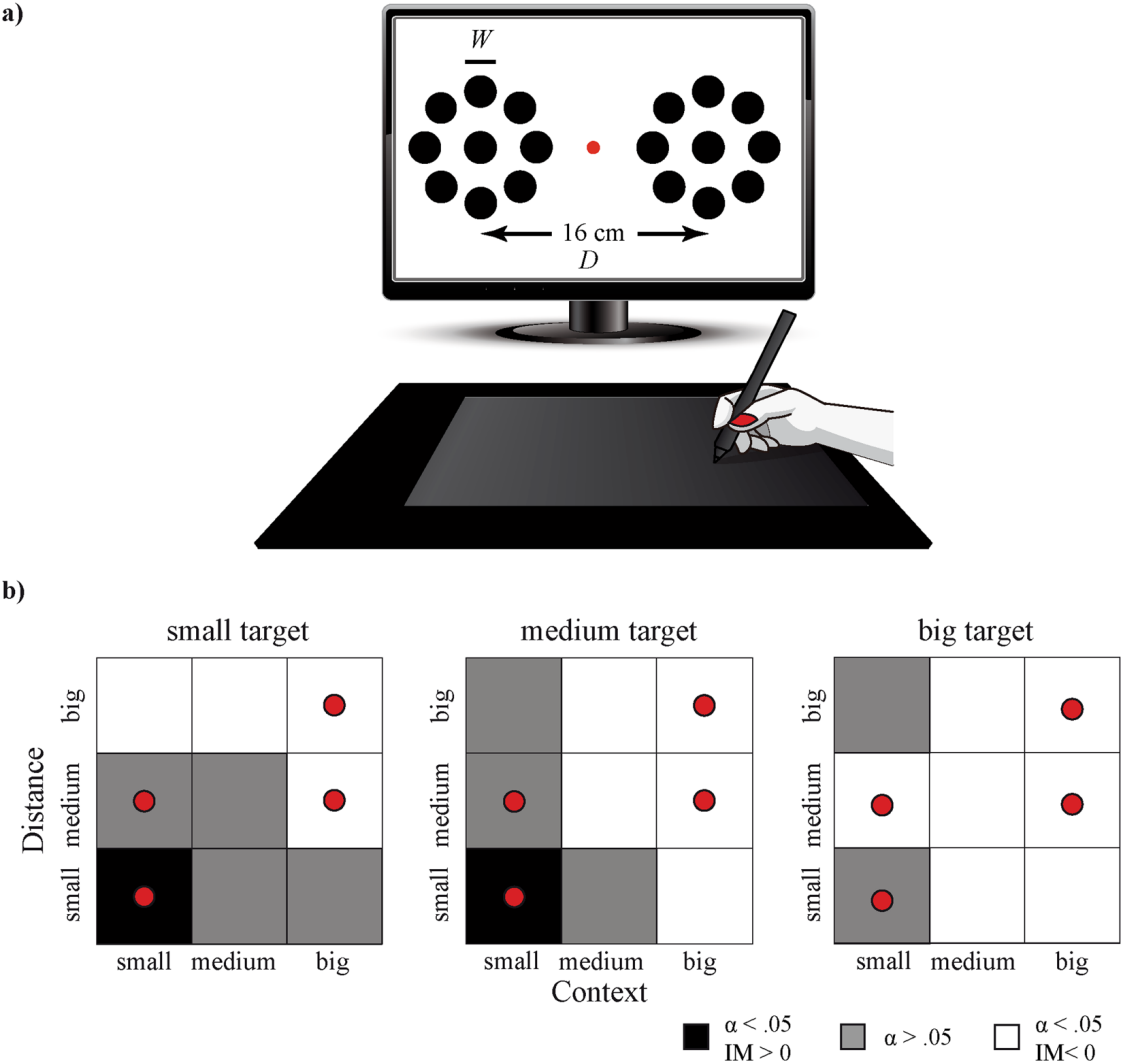
(**a**) Experimental set-up of the horizontal sliding task, with *W* representing the target size and *D* the distance between the targets on the left and right side. (**b**) Selected parameter combinations adopted from ref. 27 are marked with a red dot for each quantified target size, context size, and target—context distance. The black and white squares indicate a significant illusion magnitude (IM) for bigger perceived targets and smaller perceived targets, respectively. The grey squares show conditions that were not significantly different from the control trials (*α* = 0.05; see ref. 27 for details).

Participants read instructions on the screen, which explained that they were required to slide with the stylus as fast as possible between two targets (i.e., the centre of the left and right figure in Fig. 7). After a familiarization phase in which the participants performed up to 5 sliding movements on the tablet between two plain target circles, the participants performed two trials of 25 reciprocal movements per condition. The conditions consisted of an identical Ebbinghaus figure on the left and right side of the screen with one out of three possible target sizes (i.e., 5, 10, or 20 mm). Each target size was combined with a small or big context of target-surrounding circles displayed at a small/medium and medium/large distance between target and context circles, respectively (indicated with a red dot in Fig. 7b). To balance the factorial design, the small/medium and medium/large target—context distance combinations were grouped into relatively small and big distance between target and context circles, after verification whether this grouping changed the outcome. This resulted in 12 conditions (i.e., 3 target sizes × 2 context sizes × 2 target—context distances) for the Ebbinghaus figure. Three control conditions were added, in which the three plain target sizes were presented, that is, without context circles. Participants were asked to take breaks after each block of five trials but could also take a break whenever they felt it was necessary. The order of trials was randomized. After each trial, the participants got feedback on the number of errors to emphasize the importance of the precision.

### Movement parcellation

**Figure 8:**
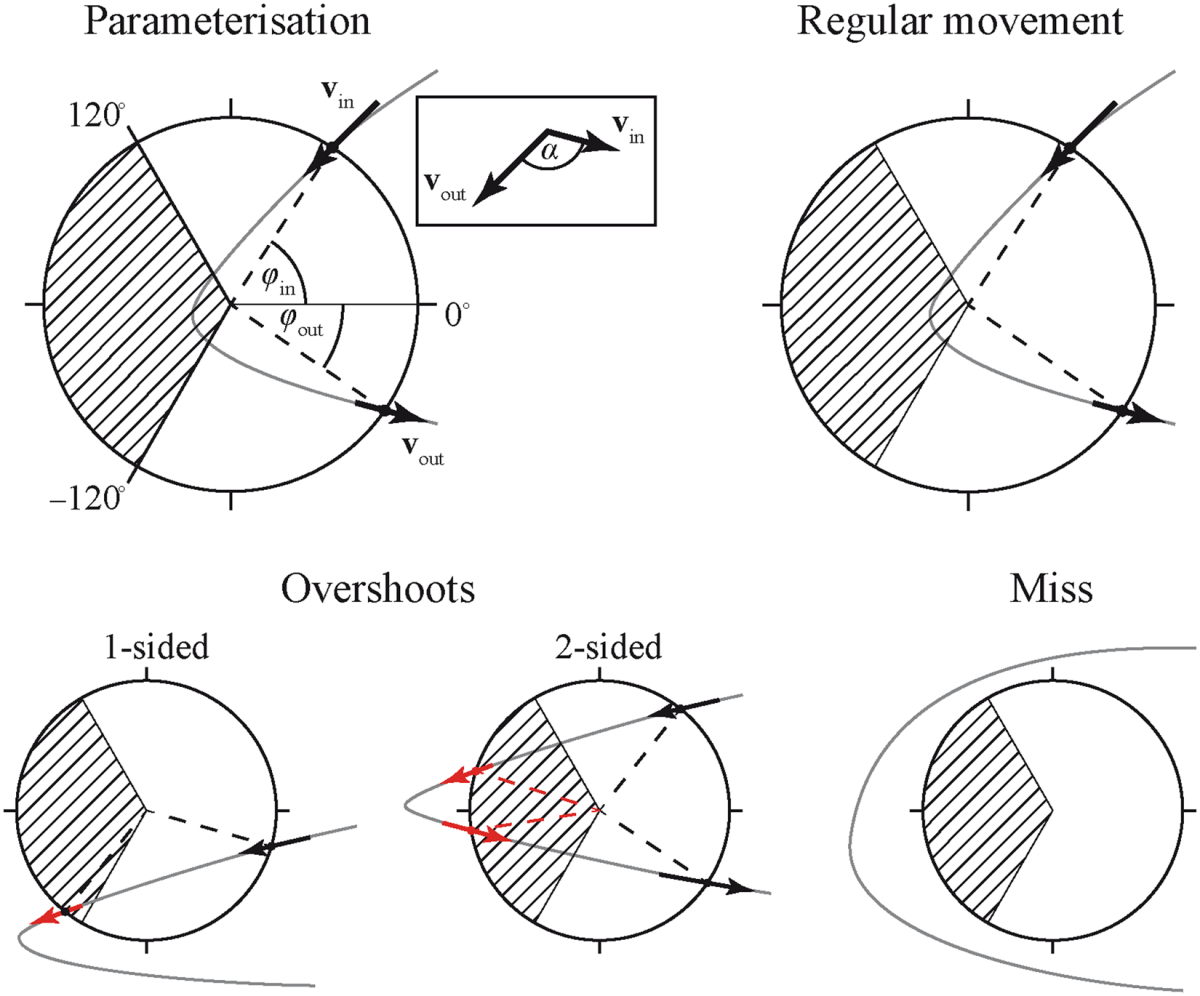
Geometrical movement parcellation and identification of the targeting phase, illustrated for a right to left movement. *V*_in_ and *V*_out_ represent the vectors and the entry and exit point, respectively, with α being the angle between the vectors. The angle between of the radius at 0° and the radius to the intersection of the movement with the target (*φ*_in_ and *φ*_out)_ should not exceed 120°. The shaded area is the area with *φ*_in_ and/or *φ*_out_ being bigger than 120 degrees. A black arrow signifies a correct entry or exit, whereas a red arrow represents an entry or exit that did not meet the requirements. Errors were identified as 1-sided overshoots if a least one entry or exit was exceeding the requirements. 2-sided overshoots had at least 2 entries and 2 exits. Misses did not have any intersections with the target.

**Figure 9:**
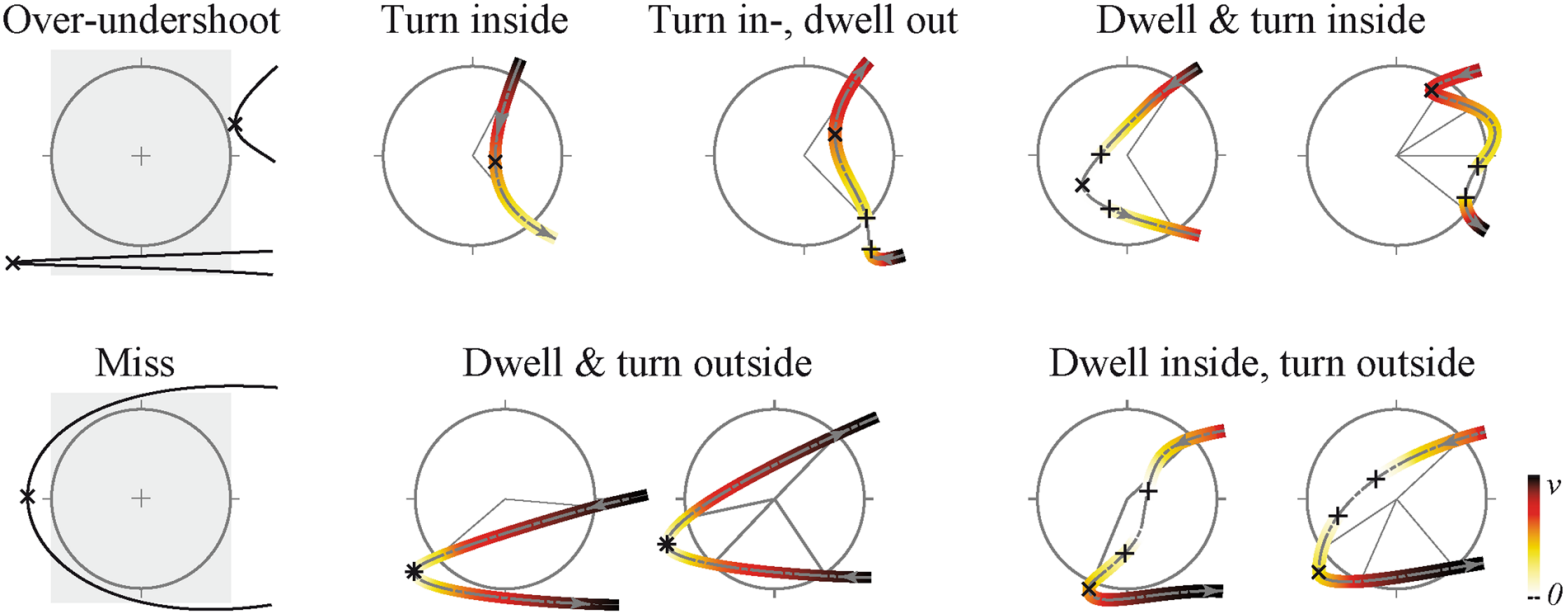
Subdivision of overshoots and undershoots and the characterization of dwell time based on the velocity profiles. The color-coding signifies the speed along the trajectory. The cross (*x*) indicates the turning point. The plus (+) indicates the movement onset and offset. The grey block for an over-undershoot and a miss represents the area that would have been included if only the horizontal position and its first derivative would have been taken into account, illustrating the possible errors that would have been falsely taken into account.

To calculate the moments of movement onset and offset, the velocity along the trajectory of the movement and its position data (*x*, *y*) on the plane of the tablet were analysed. The position data (*x*, *y*) were analysed with the aim to restrict the analysis to the horizontal plane (*x*). This movement parcellation allowed to classify and to quantify the movement errors, and to assess whether the Ebbinghaus illusion affected these measures. Fig. 8 shows the analysis of the movement in a target (in this case, the left target), in which valid movements are defined as those in which the angle between the ingoing and outgoing intersection points of the line (*φ*_in_, *φ*_out_) was smaller than 120 degrees, and the angle (*α*) between the vectors at the intersection points with the target border (*V*_in_, *V*_out_) exceeded a threshold of 42°. Note that these valid movements have one target ‘entry’ and one target ‘exit’ (*φ*_in_,). One-sided overshoots were identified as movements with also one target entry and exit but with *φ*_in_, *φ*_out_ and/or *α* exceeding the corresponding threshold. Two-sided (or *n*-sided) overshoots were characterized by at least two entries and exits. Misses did not cross the target. Misses were further subdivided into undershoots and overshoots, based on the horizontal position and the movement velocity (see Fig. 9).

Dwelling in the target occurred if the velocity profile showed a significant minimum inside the target (i.e., along the trajectory and not touching the target border). The minimum was significant if this period was drawn from a different distribution than the movement inside and away from the target. This was tested by a two-sided Kolmogorov-Smirnov test. Irrespective of the presence of dwelling, we determined the turning point inside the target (with respect to the *x*-direction). Note that both dwelling and turning can coincide.

### Dependent measures

Movement time (*MT*) was defined as the difference between movement onset and the subsequent movement onset (see Fig. 9). The ratio between the acceleration time and the movement time (*R*_*AT*/*MT*_) was quantified as the time from movement onset to peak velocity (i.e. acceleration time, *AT*), divided by the movement time. The deceleration time (*DT*) signified the time from peak velocity till the subsequent movement onset. The perceptual illusion magnitude (*IM*) was retrieved from ref. 27 and correlated with the *MT*. The misses and overshoots (one-sided and two-sided, see Fig. 8 and 9) were counted, and their sum reflected the number of invalid movements.

The probability to find a specific point at a given time in state space (*x*, d*x*/d*t*) relative to its previous state at time *t*_*0*_ is reflected by the conditional probability distribution. The conditional probability serves as a basis for the construction of vector fields (i.e., the deterministic dynamics). To compute the probability *P*(*x*, *t*) and the conditional probability, position time series were low-pass filtered using a fourth-order Butterworth filter, with a cut-off frequency of 10 Hz. The *x*(*t*) and d*x*(*t*)/d*t* time series were normalized to unity, ranging between [−1, 1]. The probability distributions were computed by concatenating all aiming movements of all participants in each condition using a grid of 31 by 31 bins. Difference probability distributions were calculated as the difference between the probability distribution of the small context condition and the big context condition. The probabilities were normalized. Kramers-Moyal coefficients (i.e., to reconstruct the deterministic part of the dynamics) were computed, which allowed for the computation of angle *Θ* between each vector and its neighbors. Subsequently the angle with the biggest value *Θ*_max_ was retained. The biggest *Θ*_max_ around the target (7 × 5 bins around the target location) was taken as a measure for the existence of a fixed point (fixed points were identified as *Θ*_max_ > 90°^33^).

One principal component analysis was performed to investigate endpoint variability as a measure of targeting precision. To test the distribution of endpoints, the principal orthogonal eigenvectors were retrieved. To test whether the distribution of endpoints got more or less dense through the experimental conditions, the endpoint distribution (*EPD*) was calculated as the range between the minimum and maximum value of the data, after outliers were excluded according to equation 1:

1 $$({Q}_{1}-1.5\,IQR)\le data\le ({Q}_{3}+1.5\,IQR),$$

In which IQR is the interquartile range (from 25% to 75%), *Q*_1_ is the first quartile (25%) and *Q*_3_ is the third quartile (75%). The *EPD* was calculated relative to the *EPD* of the control condition (100%).

### Statistics

Repeated measures analyses of variance (ANOVA) were performed on the normal distributed data, with *ID*, target-context distance, and context size as within participants factor. If significance levels were met (*α* = 0.05), the tests were followed up by Bonferroni post-hoc tests. The degrees of freedom were corrected according to the Greenhouse-Geisser method to control for non-sphericity of the data if necessary. If this was the case, the degrees of freedom were reported. Whenever the data was non-normal distributed, a non-parametric Friedman test was performed. If the Friedman test showed significant differences, the test was followed up with the Wilcoxon signed rank post-hoc tests with Bonferroni correction (α/number of comparisons). Pearson correlation coefficients were calculated to investigate potential linear correlations between the perceptual *IM* and the *MT*. Linear regression analyses were applied on the *MT* data to investigate the slope and intercept.

## Electronic supplementary material

Supplementary Materials - Movement parcellation and regression results
