# Supplementary material for: Ebbinghaus figures that deceive the eye do not necessarily deceive the hand
Source: Sci Rep. 2017 Jun 8;7:3111. doi: 10.1038/s41598-017-02925-4 (PMC5465067; doi:10.1038/s41598-017-02925-4)
Supplement: Supplementary file 1 — Supplementary Materials - Movement parcellation and regression results [file 41598_2017_2925_MOESM1_ESM.pdf]

Ebbinghaus figures that deceive the eye do not necessarily deceive the hand – SUPPLEMENTARY INFORMATION

Hester Knol[1], Raoul Huys[2,3], Jean-Christophe Sarrazin[4], Andreas Spiegler[5], Viktor Jirsa[5]

[1]Aix Marseille Université, CNRS, Institut des Sciences du Mouvement UMR 7287, Marseille, France

[2]Centre de Recherche Cerveau & Cognition, Université Paul Sabatier, Université de Toulouse, Toulouse, France

[3]CerCo, CNRS UMR 5549, Toulouse, France

[4]ONERA, Systems Control and Flight Dynamics Departement, Salon de Provence, France

[5]Aix Marseille Université, INSERM, INS, Inst Neurosci Syst, Marseille, France

Corresponding author:

Hester Knol

Aix-Marseille Université

CNRS, Institut des Sciences du Mouvement UMR 7287

13288, 13009, Marseille, France

Email: hester.knol@gmail.com

SUPPLEMENTARY MATERIAL – Evaluation of classified movements

Since the distribution of the classified movements (i.e., misses, two-sided and one-sided overshoots) is non-normally distributed, we performed a Friedman test instead of the parametric repeated measures ANOVA.

Misses

The number of misses neither showed significant differences for the left target and right target nor for any of the tested conditions.

Two-sided overshoot

On the left and right side, there was a significant influence of the experimental conditions on the number of 2-sided overshoots (left target: χ^2 (23)=87.62, $p < .001$; right target: χ^2 (23)=99.99, $p < .001$). Posthoc tests, using Wilcoxon signed-rank tests with the Bonferroni correction (α/number of comparisons), showed a significant difference between the small and medium ($Z_{left} = 3.79$, $Z_{right} = 4.60$, $p<.001$), medium and big target size ($Z_{left} = 4.53$, $Z_{right}=5.48$, $p <.001$), and small and big target size ($Z_{left} = 6.01$, $Z_{right}=6.44$, $p <.001$), for both the left and right target (see Fig. S1). No significant effects were found for repetition, context size, and the distance between the target and context.

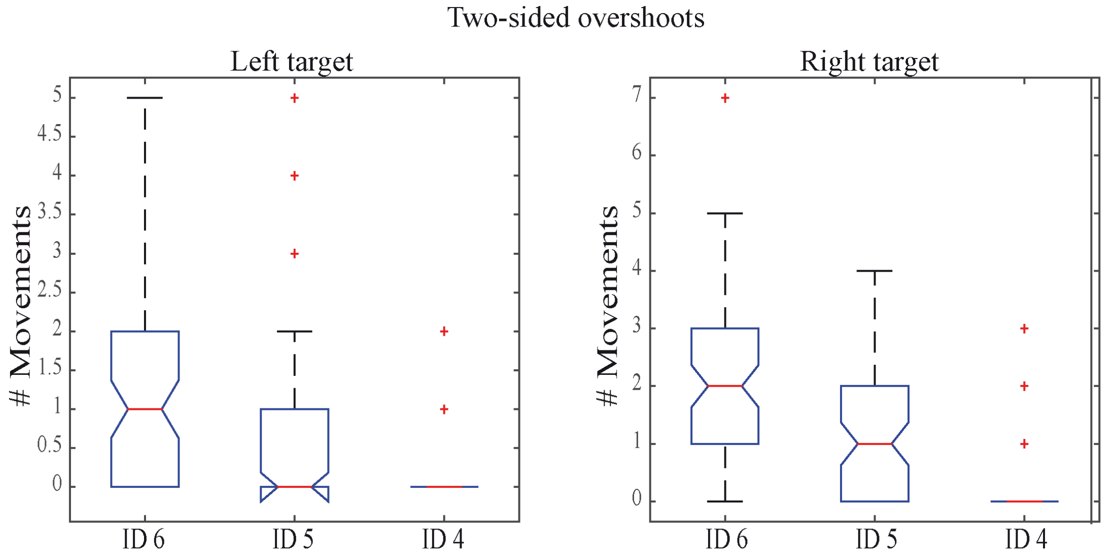

Figure S1. Box plots of the number of two-sided overshoots for ID 6, ID 5, and ID 4 for the left and right target.

One-sided overshoot

On the left and right side, there was a significant influence of the conditions on the number of 1-sided overshoots (left target: χ^2 (23)=43.65, p < .01; right target: χ^2 (23)=37.73, p < .05).

Posthoc tests showed a significant difference between the small and medium (Z_{left} = 3.96, p< .001, Z_{right} = 3.30, p= .001), and small and big target size (Z_{left} = 3.39, Z_{right}=3.89, p <.001), for both the left and right target (see Fig. S2).

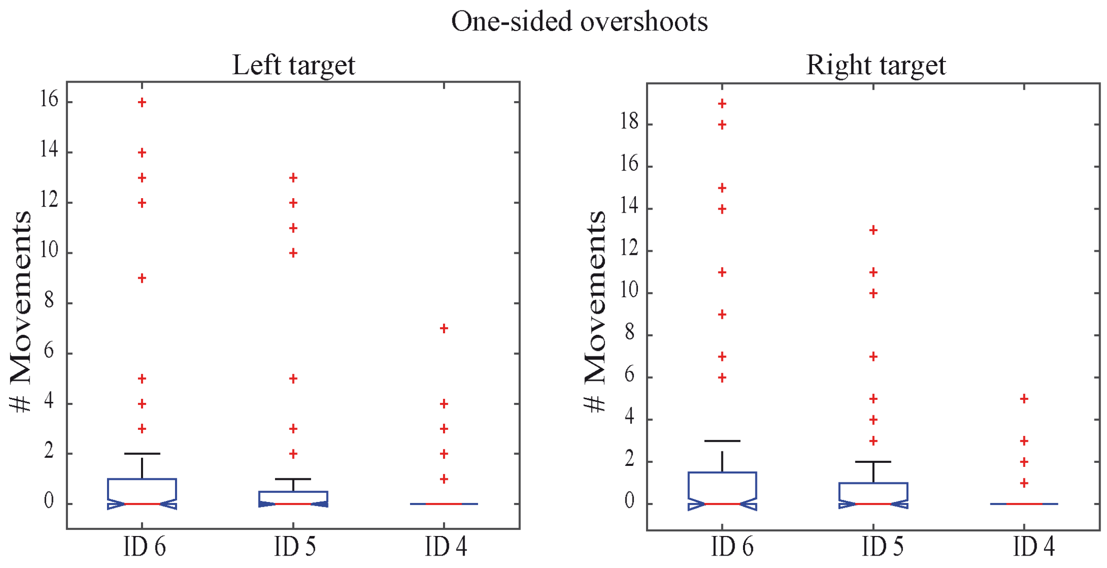

Figure S2. Box plots of the number of one-sided overshoots for ID 6, ID 5, and ID 4 for the left and right target.

Invalid movements

The sum of the misses, the one-sided and the two-sided overshoots represents the number of invalid movements for each experimental condition. On the left and right side, there was a significant influence of the conditions on the number of invalid movements (left target: χ^2 (23)=83.71, p < .001; right target: χ^2 (23)=112.17, p < .001). Posthoc tests showed a significant difference between the small and medium (Z_{left} = 4.51, Z_{right} = 4.38, p<.001), medium and big target size (Z_{left} = 4.20, Z_{right}=5.39, p <.001), and small and big target size (Z_{left} = 6.04, Z_{right}=6.61, p <.001), for both the left and right target (see Fig. S3).

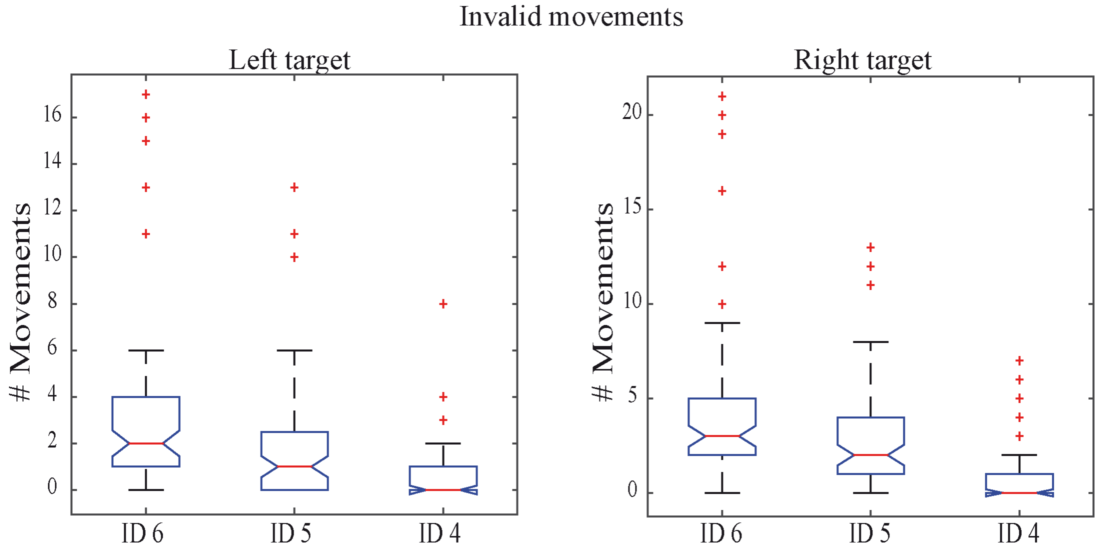

Figure S3. Box plots of invalid movements for ID 6, ID 5, and ID 4 for the left and right target.

Trajectory Length

The length of the trajectory l is obtained by calculating the Euclidean distance, that is, $l[n, n + 1] = (x[n + 1] - x[n])^2 + (y[n + 1] - y[n])^2)^{1/2}$ of pairs of successive samples $s[n]$ at time point n and $n+1$ on the plane ($x[n]$ and $y[n]$), and then calculating the sum $l = \sum_{n=1}^{N-1} l[n, n+1]$, where N is the total number of sampled points in the xy-plane. There was no difference between the trajectory length from left to right or right to left (from start to end) (t-test, p=.95). Furthermore, there was no significant effect of target size on the trajectory length in the control condition (χ^2 (26)=2.89, p = .24). Further analysis of factors context size, target—context distance, and trial number showed no effects on the trajectory length.

X-Y separation

To check whether the X-Y plane can be reduced to just the horizontal direction (X), we checked whether a scale separation occurred, with the criterion that the Y direction contributed more than 10% to the total trajectory length.

In 4.3% of all movements, the Y direction contributed more than 10% to the total movement length as compared to the X direction.

Linear Regressions

Linear regression analyses were performed on all data points at group level and individual level. The results of the linear regression analysis are shown in Table S1.

**Participant**	**R^2**			**$RMSE^2$**			**a**			**b**		
	**C**	**S**	**B**	**C**	**S**	**B**	**C**	**S**	**B**	**C**	**S**	**B**
**1**	0.72	0.76	0.72	0.01	0.01	0.01	-0.25	-0.33	-0.14	0.21	0.24	0.20
**2**	0.57	0.60	0.60	0.02	0.02	0.02	0.02	-0.09	-0.04	0.19	0.21	0.21
**3**	0.81	0.83	0.78	0.01	0.01	0.01	-0.51	-0.60	-0.46	0.27	0.29	0.26
**4**	0.73	0.79	0.72	0.02	0.01	0.02	-0.48	-0.57	-0.49	0.27	0.28	0.27
**5**	0.72	0.79	0.75	0.06	0.05	0.03	-0.82	-1.16	-0.41	0.47	0.53	0.38
**6**	0.75	0.79	0.73	0.02	0.01	0.02	-0.49	-0.29	-0.25	0.30	0.26	0.25
**7**	0.76	0.74	0.77	0.01	0.01	0.02	-0.46	-0.43	-0.59	0.26	0.25	0.30
**8**	0.68	0.67	0.67	0.01	0.01	0.01	0.13	0.10	0.14	0.16	0.17	0.17
**9**	0.75	0.77	0.58	0.03	0.03	0.04	-0.67	-0.69	-0.25	0.36	0.36	0.30

Table S1. Linear regressions' estimates for individual movement time data over the index of difficulty, with *a* representing the intercept, and *b* the slope for the control (C), small context (S), and big context condition (B).

Frequently the linear regression analysis is performed over mean movement time data, leading to higher R^2 values and a smaller $RMSE^2$. For completeness the results of the linear regression analyses for the control condition (C), the small context (S), and the big context (B) are reported in Table S2.

**R^2**			**$RMSE^2$**			**a**			**b**		
**C**	**S**	**B**	**C**	**S**	**B**	**C**	**S**	**B**	**C**	**S**	**B**
0.9974	0.9939	0.9997	<.0001	0.001	<.0001	-0.39	-0.45	-0.28	0.28	0.29	0.26

Table S2. Linear regressions' estimates for group mean movement time data over the index of difficulty, with *a* representing the intercept, and *b* the slope for the control (C), small context (S), and big context condition (B).
